# Emotional constraint, father-son relationships, and men's wellbeing

**DOI:** 10.3389/fsoc.2022.868005

**Published:** 2022-09-13

**Authors:** Anne Cleary

**Affiliations:** UCD Geary Institute for Public Policy, University College Dublin, Dublin, Ireland

**Keywords:** masculinity, emotions, emotional constraint, suicide, men's health, fathers, fathering, childhood trauma and adversity

## Abstract

Male rates of suicide exceed female rates and research findings indicate an association between particular practices of masculinity, specifically emotional constraint, and male suicide. This paper examines gender and family influences on men's wellbeing, based on in-depth interviews with a sample of fifty-two men, aged 18–30 years, who made a clinically serious or near-fatal suicide attempt and were recruited following presentation to hospital. Themes derived from the analysis included *learning about masculinity* which relates to the gender culture within the home, *the regulation and enforcement of behavior by peers* and *father-son relationships*. Results demonstrated that the men were generally from families where hegemonic ideals of masculinity, emphasizing strength and emotional stoicism, were practiced. This gender environment, which was reinforced in the neighborhood, restricted behavior and the expression of feeling, shaped communication between fathers and sons and affected the father's ability to emotionally engage with his son. Fathers were significant figures in these men's lives and were role models for demonstrating masculinity practices but there was an absence of positive, nurturing, relationships between fathers and sons and this influenced the son's gender learning and his wellbeing. Fathers who were emotionally distant, and particularly those who were abusive, gave rise to feelings of rejection, sadness and anger in their sons but problematic father-son relationships were not addressed nor ill-treatment in childhood disclosed due to gender-related constraints on expression. Restrictions on expression and prohibitions on revealing weakness denied the men a space to explore as well as manage the issues of their lives and prevented them from revealing distress. They coped by sublimating problems and disguising vulnerability and by seeking emotional comfort within intimate partnerships but these men were susceptible to situations which threatened their psychological security. Overall, the study demonstrated challenges for males raised in settings of hegemonic masculinity and the importance of nurturing father-son relationships for male wellbeing. The results imply the need for a focus on the benefits of positive fathering and the inclusion of more nuanced messaging relating to men's emotions in Public Health messaging.

## Introduction

High levels of male, compared to female, suicide exist in Europe and the United States although male suicide rates vary across as well as within countries, based on cultural and socioeconomic factors (Cleary, 2019; World Health Organisation, 2014, 2019). A gender explanation has been suggested for the high prevalence of suicide amongst men in Western societies and linkages have been identified between practices of hegemonic masculinity, particularly emotional restriction, and male suicidal behavior although the detail of this association is unclear (River and Flood, 2021). The aim of this analysis is to explore how particular cultural environments affect gender and wellbeing by focusing on the influence of the family and the father on the son's practice of masculinity.

Individuals acquire ideas about gender within a particular cultural setting and this provides the broad script which the male draws from when negotiating his behavior and his emotional life. These gender concepts are introduced at an early age and generally maintained by surveillance from family and peers (Kimmel, 1994). The expression of feelings is gendered, more rigidly so in some cultural environments, reflecting a belief that men and women have different emotions, that these emotions are natural or innate and channel males and females into various forms of behavior (Shields, 2007). The regulation of emotions can impact negatively on mental wellbeing and create a risk of suicidal behavior (Cleary, 2019). The binary division of emotions is associated with practices of hegemonic masculinity, a way of performing manhood that emphasizes strength and discourages behavior or displays of feelings which imply weakness (Kimmel, 1994; Connell and Messerschmidt, 2005). Hegemonic masculinity is the idea that a dominant, socially constructed, form of masculinity is given preference over other expressions of masculinity and femininity (Connell and Messerschmidt, 2005). Men are not a unitary group and male/female differences relating to behaviors and emotions are, in reality, more fluid (Simon and Nath, 2004; Patulny et al., 2017) yet, oppositional gender concepts and unitary notions of men and male emotions underline much suicide-related research. This analysis is part of a study (Cleary, 2019), which adopted an alternative research direction, in the tradition of Douglas (1967) rather than Durkheim (1951), that sought to explore male suicide from a social constructivist perspective, by allowing the subjects to relate their own stories around suicidal action. Within these self-constructed narratives childhood, fathers, and father-son relationships featured prominently and while these topics have emerged in other work related to male suicidal behavior (Wagner et al., 2003), they remain relatively underdeveloped in the research literature. This analysis focuses on family and peer influences on gender performances, the contribution of the father to the man's practice of masculinity and the implications of these elements for his wellbeing.

The family and fathers contribute to gender learning and emotional wellbeing in their sons in various ways. Fathers are the usual gender models for young males and the way the father engages with his son and demonstrates masculinity influences both the son's gender-related behaviors and his wellbeing (DeFranc and Mahalik, 2002; Adamsons, 2013). The father's involvement and his ability to develop a nurturing relationship with his son has important consequences for the child's psychological development (Lamb, 2010; Adamsons and Johnson, 2013) and problematic father-son relationships can have negative psychological implications for males (Wagner et al., 2003; Videon, 2005; Bronte-Tinkew et al., 2006). Males who have close relationships with their fathers are more likely to be open about problems and to develop enduring relationships and friendships with others, including other males, while negative father-son interaction is associated with deficits in forming relationships, poor self-image and psychological problems including suicidal behavior in males (Gould et al., 1996; Fergusson et al., 2000; DeFranc and Mahalik, 2002; Johnson et al., 2002; Wagner et al., 2003). Fathers with traditional views of gender tend to have less positive relationships with their children (Smyth and Russell, 2021) and fathers who adhere rigidly to hegemonic masculine values are less likely to have close relations with their sons (DeFranc and Mahalik, 2002). Moreover, the parenting styles of fathers tend to be transmitted to sons (Brown et al., 2018; Jessee and Adamsons, 2018). The gender and relational environment within the home and specifically father-son relationships are therefore relevant for the growing boy's wellbeing and the existence of difficulties within the family, such as physical and or emotional abuse, adds complexity to this situation.

Childhood trauma appears to have a particularly negative impact on males as the risk of suicide is higher for males than females in these circumstances (Dube et al., 2001; Wagner et al., 2003; Afifi et al., 2009; Weich et al., 2009). Those who die by suicide are significantly more likely to have experienced ill-treatment and traumatic events in childhood and the probability of suicide increases with the nature and extent of the negative experiences (Dube et al., 2001; Molnar et al., 2001; Enns et al., 2006; Seguin et al., 2011; Giupponi et al., 2018). Children who face adversity usually experience distress but long-term psychological outcomes are dependent on the ability of the child to access support (Dube et al., 2001). Gender cultures which feature emotional restriction for males may prevent disclosure of these experiences and there is evidence to support this from studies which show that males who suffer childhood abuse commonly use coping strategies such as denial and emotional suppression, misuse alcohol and drugs (Afifi et al., 2009; Hughes et al., 2017) or become involved in violence (Moreira et al., 2020). Males raised in traditional or hegemonic home environments have a higher risk of suicide (Afifi et al., 2009) and it appears that men in working-class settings are more likely to affirm this type of masculinity (Joe and Kaplan, 2001) and manage their behavior and emotional performances in line with hegemonic practices (Cleary, 2012, 2019; Mikorski and Szymanski, 2017). Working class males have comparatively high rates of suicide and studies have shown how economic disadvantage contributes to suicide risk *via* higher exposure to adversity (Turner et al., 2006) and reduced life options (Redley, 2003). In this paper I explore how gendered environments, negative relationships with fathers and traumatic experiences affected the lives of men who attempted suicide.

## Methods

The aim of this research study was to understand suicidal action from the perspective of men who attempted to take their own lives and to explore the background circumstances and motivations involved. This social constructivist approach was prompted by the author's theoretical position and by a relative scarcity of data based on this method. The focus of this paper is to explore the impact of exposure to hegemonic ideals and practices, including restricted emotional expression, on men who made a suicide attempt in adulthood and the influence of fathers and father-son relationships within this gender scenario. The analysis is based on interviews with fifty-two men who made a clinically serious or near-fatal suicide attempt.

### Sample and data collection

Inclusion criteria included gender (male), age (18–30 years) and high level of severity and intent in that all those included in the study had made a serious suicide attempt with definite intent to die. The age and gender criteria were chosen to reflect a population group with a high rate of suicide in Ireland and other Western countries. The interviewees consisted of a consecutive sample of 52 men from three hospitals in the Dublin area and involved all those who presented to these hospitals over a specific time period who fulfilled the study criteria—males aged 18–30 years who had made a clinically serious suicide attempt. Two of the hospitals are district hospitals (with major Accident and Emergency and psychiatric units) and the third a psychiatric unit, which admits patients from a nearby general hospital (the hospitals cannot be named due to confidentiality requirements). The sample can be regarded as representative due to the seriousness of the attempts and because these hospitals were likely to receive all such admissions from this area over the period of the study. Participants were referred by the liaison psychiatrists working in the Accident and Emergency Departments of the hospitals. One man refused to be interviewed. The high response rate was achieved, I believe, primarily because these men wanted to talk about this critical event in their lives and I presented an opportunity for them to do so. A common reaction from the men when asked to participate was that they were glad to do so in order to help others in similar circumstances. There was strong support from hospital personnel for the research and clear lines of communication established (including a designated contact person in each center) prior to commencement of the study. This followed extensive negotiations with the hospitals relating to ethical, access, and procedural issues prior to permission being granted for the research project. The fact that I was a woman may have been a positive factor as it emerged that, for these men, speaking about personal matters with a female was not as proscribed as relating in this way to a male. In addition, I was an experienced interviewer, able to listen and cope with strong emotions as well as silences. I was also genuinely interested in what they had to say.

The participants were interviewed, by the author, as soon as possible after the suicidal action, depending on the extent of their injuries. An unstructured interview schedule was used in the session consisting of one introductory question “Can you tell me how you came to be admitted here?” Thereafter, no further pre-set questions or topics were covered but prompts were used and questions asked in response to issues raised by the respondent. This approach was adopted in order to avoid pre-categorization of the suicidal action or motives and to allow the respondent to tell his story in his own way following in the tradition of Douglas' (1967) work. A common feature at the start of the interviews was a verbal outpouring after this initial question and in almost all cases they developed the narratives themselves and required little prompting. Common topics emerged which was understandable as they shared a very specific experience, i.e., the suicide attempt. Their initial narrative was usually about the lead-up to this event and this generally led on to background and family issues. The theme of fathers and relationships with fathers, which is a focus of this paper, emerged spontaneously and was not included on any topic. Prompting when it occurred was usually quite general, for example, “tell me about that” when they raised an issue (there are examples in the current text of these questions and prompts), and as the fieldwork progressed I asked a small number to tell me about their family if they had not mentioned this. Interviews lasted ~1 h, but some were considerably longer and were audio-taped. All but two men agreed to this and in those cases I took notes which I wrote up following the interview. I also recorded notes and observations on the interviews after each session including tracking my response to the session and the subject. The majority of the interviews could be classified as successful encounters as there was a good level of engagement between us and the respondent appeared relatively relaxed but this was not always so. Two interviewees became hostile as the sessions progressed and there were instances when distasteful opinions or violent actions were described which were difficult to listen to. The interviewing style was informal with an emphasis on listening but this does not imply uncritical acceptance of their accounts. My position was that of an academic researcher from a university and I stated this verbally and in the consent form as well as emphasizing that I had no connection with the study hospitals.

### Data analysis

The interview tapes and field-notes were transcribed and the analysis carried out *via* computer and manual methods. The data were analyzed using a modified version of grounded theory (Corbin and Strauss, 2014) guided by Douglas's (1967) methodological approach to this topic of enquiry and Connell's (2010) life history analysis. I listened to the tape following the interviews and read all transcripts and field-notes on an ongoing basis to obtain a comprehensive picture of the data. From the beginning, narrative patterns emerged—not surprisingly related to explanations for the suicidal behavior and the way in which the suicidal pathway developed. Other themes which emerged concerned family background and relationships, enduring emotional anguish and restrictions on disclosing distress. I then used a computer program for qualitative data analysis (NUD^*^IST) to identify frequently occurring words and phrases linked to these themes. I also produced a summary note relating to each participant based on the transcript and field-notes. Following this I re-read the transcripts, field-notes and individuals' summaries continually to establish themes. I then examined relevant literature and moved back and forth between the literature and transcripts to develop the thematic analysis. Theoretically the analysis was driven by a social constructionist framework and by the work of masculinity writers such as Connell (2005, 2010). The present paper focuses on topics relating to family background and relationships with fathers which represent a re-analysis and elaboration of issues already reported (Cleary, 2019). Three main themes were derived from this re-analysis of the data. The first theme (*learning about masculinity*) relates to the culture of masculinity the men were exposed to growing up, the second theme (*the regulation and enforcement of behavior by peers*) concerns the influence of neighborhood and peers and the third theme (*father-son relationships*) refers to father-son interaction and is divided into subthemes, *seeking love and care from the father, rejection by the father* and v*iolent fathers*. In writing up I have referred to the men's life stories and have used verbatim quotations to stay as close as possible to the meanings contained in their own accounts.

### Ethical considerations

Approval for the study was obtained from the hospitals' ethics committees. Before referral, the man was informed about the study, advised that the project was independent of the hospital and his treatment regime and that participation was entirely voluntary. When I met the potential participant, I explained the nature of the study in greater detail, answered any questions and again emphasized the voluntary, confidential, and independent (of treatment) aspect of participation. After this process was completed the man signed a form, developed in co-operation with the Ethics Committees, giving his consent to participate in the study. Participants therefore had a number of opportunities to decline an interview which represented an important safeguard for them at a vulnerable time. The data was anonymised, cleaned of all identifying features, before it was removed from the hospitals and the list of participants, to which only the author had access, was kept in a locked environment in the university and destroyed at the required time. Pseudonyms are used in this paper and all identifying features have been removed.

## Results

The aim of this study is to explore the impact of exposure to hegemonic ideals and practices, including restricted emotional expression, and the influence of fathers and father-son relationships within this gender scenario. The first theme (*learning about masculinity*) derived from the data concerns the culture of masculinity and emotional expression these men were exposed to growing up which shaped their ideas about gender and indicated acceptable practices for males. A related theme (*The regulation and enforcement of behavior by peers*) deals with the influence of neighborhood and peers in terms of conformity to hegemonic practices. The third theme considers the influence of the father on the man's practice of masculinity and the implications of father-son relationships for both gender performance and wellbeing and this is subdivided into key aspects of father-son relationships which derived from the data- *seeking love and care from the father, rejection by the father* and v*iolent fathers*.

### Learning about masculinity

The men in this study were mainly from lower socioeconomic backgrounds and the home was an important site for acquiring ideas about masculinity and receiving guidance on how to behave and to express oneself. Fathers were significant figures within this system and in the men's lives, whether fathers lived within or outside the home, and were often cited as role models or reference points against which the men compared their own performance of masculinity.

*My dad is everything to me. He's one of those people really I can look up to. He made himself into what he is and he's just great. …God knows I've tried but I'm not his son at all, because his son would be able to go out and do that. That's what my father would do, not what I'm doing. …You follow in your father's footsteps and you'll be better. If I was to follow his footsteps, be the man he is, be the husband that he is to my mother and be the father that he is to us I would be… …that's what you want and it's a horrible thing when you want something and you can't have it*. Alex

The form of masculinity evident in their families was generally traditional or hegemonic in terms of values and behavior and the father tended to occupy an authoritarian position in the household.

*He was a hard man to grow up with. He was strict, always had that in him. If you crossed him obviously you paid the price*. Matt

The father was instrumental in imparting and enforcing ideas about masculinity and, in line with hegemonic ideals, sons were guided from an early age to avoid signifiers of vulnerability.

*I learned from an early age from my father that having problems is not a good thing to have. Well, just things that happened to me. I remember one time I got moderately upset about something and my mother and my mother's friend were being all mollycoddling to me and my father thought this was absolutely a big laugh, disgraceful, so he just gave me a look of severe disgust and embarrassment that I had been allowing myself to be mollycoddled by females. I was about eight or something*. Nicholas

As Nicholas implies, there was an awareness from a young age of distinct behavioral and expressive norms for males and females and that feelings had to be managed according to these binary gender lines. Interaction between males in the household differed from that between mothers and sisters and mothers overwhelmingly did the emotional work in families. Fathers were generally viewed by participants as emotionally distant and communication between males in the household rarely diverged from discussion of impersonal topics. Matters outside this narrative sphere were ignored or dealt with *via* teasing or “slagging” which the participants recognized as a way of avoiding emotional intimacy and uncomfortable topics. Gender conformity was expected and behavior set out by the father was reinforced by other males within the family as demonstrated by Alex's account of interaction within his family.

My brothers tease me and all like that. In a way they upset me because me da says “well they're your brothers and that's what your brothers do”. But yeah, they're my brothers but they know it upsets me. I generally really just can't take a joke. I could before but now it's like I'm looking for an excuse just to show (anger). …I talk to my brothers and I talk to my da. They thought I was just being too much of a whinger which I was. I was still whinging about it. They were joking and having a laugh but they didn't really realize the extent that I don't want to deal with that. They are supposed to love me. And they do. I know they do but I was twisting it and everything. I was trying to make something bad out of it.

***When you told your dad about the teasing, what did he say?***

“Stop whinging”. Stop your moaning about it and he was right and I should have because they all mess with each other. …We're a very close family and that's what makes it very hard as well. I think the fact that we're so close makes it really hard. Because we're so close you hurt people even more.

There was generally no outlet within the home to discuss feelings outside these gendered parameters and signifying weakness was discouraged. Even when vulnerability was uncovered it did not appear to disrupt these practices of masculinity. Leo, who was the youngest in the family, portrayed his relationship with his father as emotionally distant (“more based on respect than love”) and described his older brothers as “tough men” who personified strength. Communication between the male members of Leo's family was, as elsewhere, restricted to discussing practical matters and to “kidding around” which emphasized Leo's sense of difference.

*It's hard to live up to your brothers. I felt that I didn't really belong. I don't know. I can't talk to them at all. I have said it to them. I don't know my brothers the way I should know them. Anytime we see each other we always start messing. No one is ever serious*.

In the aftermath of Leo's suicide attempt a brother revealed that he suffered from depression and Leo's response to this disclosure, and to his brother's attempt to engage authentically with him, was to redefine this sibling as “different” and the narrative contains elements of vulnerability and strength, as noted in other accounts of male communication (Schwab et al., 2016).

*One of my brothers, he came in and he said “what's wrong with you” and he started crying and I started crying as well. He's kinder with me in a sense and I put it down to him not being a hard chaw. All my brothers are big and he's not big. That's what I think. And he started crying and I started crying and he said “just come on, we'll talk about it, it'll be alright” and I just told him “it's hard enough but you get through it”*.

These stories illustrate the existence of hegemonic practices including the regulation of feeling in the participants' homes. These masculinity ideals were contested but complete rejection of the prevailing masculinity was difficult due to expectations within the home and enforcement in the neighborhood. This could lead to feelings of helplessness as in Leo's case. He sought a very different masculine identity from his father and brothers but felt he lacked the educational and economic resources to pursue another way of life and was aware of the consequences of deviating from local gender norms as he had been victimized in school.

*It's like being trapped. Did you ever have that feeling that like you felt you didn't really belong where you are? It's kind of like that. Sounds weird but maybe I shouldn't have been born or something*. Leo

### The regulation and enforcement of behavior by peers

Expectations relating to male behavior and expression existed within the family but practices of hegemonic masculinity were rigidly enforced outside the home.

*So you just get shunned if you're different. That's being a fag. When you're growing up there's a lot of pressure not to be gay. …if you're gay you get an awful time*. *Life should be wife and kids, that's life. …Gay and feminine is the same. They just think that what you're wearing or the way you stand or the way you sit or your hands move when you talk that you're gay so you get punished for that in society especially when you're teenagers and I certainly did. …That's the way men are*. George

These norms were generally not endorsed by the participants but they were conscious of exhibiting acceptable markers of masculinity and having a gay identity was hazardous, evidenced by accounts of victimization and the fact that being unable to disclose one's sexuality was directly implicated in at least two of the suicidal actions. Accessing alternative masculinity sites was difficult as the majority of the men felt they lacked the economic and educational resources to do so. In general they conformed, at least superficially, by managing performances and concealing vulnerability.

*I've always grown up in a bit of a rough area, you know what I mean. My whole life has been surrounded by drugs…So you don't like to leave out, don't like to give any sign of weakness or…tell your closest friend that you might be this or you might be that, you know what I mean. People can turn and use it against you, you know that way…That's why I wouldn't say anything to anyone*. Liam

School was a key site for enforcing hegemonic masculinity and was a profoundly negative experience for many of the study participants. Almost all the men went to all-male, public (non-fee paying), schools in the locality and there were few opportunities for movement between schools. People were victimized for a multiplicity of reasons but any signifier of weakness was targeted.

*It was down to someone who wears glasses. Someone with something that is different. If you're quiet that's it, you can't be quiet. You have to be some way outstanding or you don't survive. I was picked on a lot at school. I had an awful lot of torment in school over the years. I wasn't one of the strongest boys. But as I got older obviously I got bigger and bigger and was able to fight my own battles and I had friends and that was grand then. But before that I was tormented as a kid. Primary was hard. …I always had a good friend. I always had good friends wherever I was but the bullying aspect was always there and that used to get me. I think it was just generally picking on the weaker ones*.

***Did it affect you?***

*It did yeah, that's why I'll never recover from it. The way I let it happen. It's probably up to me to face that but that's the way I let it happen. You don't feel as adequate as the others*. Matt

The consequences of bullying continued to haunt the victims long after they had left school. Victimization seemed to instill in the affected men a perception that they were weak and inept and ended the educational aspirations of people like Nicolas who had intended to go to university but were forced to leave school early ‘*because I was abused when I was in school'*. The misery caused by victimization was often compounded by family difficulties as these situations frequently made one susceptible to bullying and made it less likely that the victimization would be disclosed.

### Father-son relationships

Father-son engagement added gender and emotional complexity to the hegemonic environment these men inhabited. Relationships with fathers were a consistent theme in the men's stories, from childhood reflections to adult experiences, including encounters with fathers following the suicide attempt. Father-son relationships were almost never close, more commonly antagonistic and at times mired in violence. Conflict related primarily to the father's inability to demonstrate love and care for his son (*seeking love and care from the father*) and or to the father's abusive behavior *(rejection by the father* and *violent fathers)*.

#### Seeking love and care from the father

The love and affirmation of fathers was consistently sought by the men in this study but rarely demonstrated in an observable or consistent way and the hegemonic norms which prevailed within the home contributed to this situation. Father-son relations operated within the framework of emotional restriction described above which prevented the communication of feeling needs and this was a barrier to bonding between fathers and sons. The fact that the father was the authoritative male figure within the household added complexity to this situation and a common criticism directed at fathers was their inability to engage in a caring way while at the same time exercising power within the home.

*I don't get on with my father. I don't know. When I say I don't get on with him, I see him, I speak to him. I never talk to him. He never talked to us. He was just a normal sort of, go to work, come home, have dinner, watch a bit of telly, go to bed. I probably would have liked to have been closer to him when we were younger but it wasn't really an option. I certainly wouldn't call him warm. He was strict. I don't really remember an awful lot about him when I was younger. My father was a person who didn't show his feelings. He still doesn't even now. …It was just a normal childhood. … We had the strict side of it alright, very strict. Don't question it. If you did go against it you'd get a hiding. Not all the time, not to a serious extent. It probably seemed serious at the time. My dad used to slap us. Yeah, I was afraid of him, I suppose, in a way. If he said something you'd do it quicker than for my mam. … I suppose we were never asked for our opinion. Nothing was ever discussed. “That's what it is, take it or leave it” or you couldn't even leave it, “take it”. I'd say that is more important to children, that you talk to them in a proper manner like I talk to you or you talk to me and treat them like an adult. If you treat them like they have intelligence they'll use it at least*. Larry

Larry's resentment of his father and his behavior resulted in serious, sometimes violent, confrontations between them and influenced Larry's life choices in significant ways. At a point when he had the opportunity to pursue the career he wanted he refused a place in university when his father encouraged him to take this up and offered to support him financially.

*I had the chance but I didn't take it which didn't go down very well* (with his father). …*I was going to do* (subject named) *in* (university named) *and I had the points* (grades) *and all but at that stage I had worked about three or four months in* (mentions employment). *I came home on Tuesday night about two in the morning. It was very late anyway and my dad was there, still up, waiting up. Of course I got the letters* (from the university*) but didn't show anybody and he was there with the cheque on the table saying “you're going and that's it”. So I said “no I'm not”. So that was it, end of conversation, never discussed again. This was a person who hadn't taken an interest in eighteen years*.

In common with many of the men in this study, Larry left an unhappy home situation to form an intimate partnership in early adulthood and while he was highly critical of his father's lack of emotional engagement, he recounted a similar pattern of avoiding intimacy with his wife and children, became increasingly unhappy in his marriage and, like his father, began to drink heavily. In this way, intimate partnerships, which offered the potential for offloading painful feelings and attaining emotional security, were a risk factor for these men as the psychological liabilities they brought to the partnership often contributed to its demise and this frequently precipitated the suicide attempt. According to Larry his upbringing had not equipped him with an understanding of, nor a repertoire to deal with, his emotional needs and following two near-fatal suicide attempts (and the ending of his marriage), he decided to engage in counseling to address these issues.

Similar themes were evident in Kieran's story. He had experienced significant trauma in childhood and sought emotional comfort within an intimate partnership in early adulthood but when this ended he attempted suicide. Kieran disliked his father intensely and cited this as the reason for spending much of his early childhood in a relative's house where he was sexually abused. When the abuse become known he was taken into care and refused to confirm that the abuse had occurred as he wanted to protect the perpetrator who, according to Kieran, had provided the kind of love and care his own father had failed to give.

*I loved him. I still do and it's very hard even now, it's very hard. I really cared about him. I knew what happened was wrong. He'd be there if I was playing a football match and come up and watch me and stuff like that. Just things. …He was a very hardworking man and that and far from my dad. He'd be more like he'd look after his family better and stuff like that*.

Kieran's attitude toward the perpetrator of the abuse contrasted with the anger he directed at his father who he portrayed as an inadequate husband and father whose deficiencies were responsible for the trauma he suffered in childhood. As he relates, the relationship was antagonistic and there was no possibility of him confiding in his father about the abuse.

*I just never liked him. I never used to see that as home. …I remember one time when I was about fourteen and looking at him and saying “I'm going to get you eventually, you know that”. I hate my father, hate him. I have no feelings at all for him. Literally nothing. I hate the man. I am not a fool, I am not a hypocrite. That's just the way I feel and that's the end of it. …I was in the hospital the other night after that* (suicide attempt) *happening and I woke up and he was there and he started talking to me. I just ignored him. Even in the state I was in*.

Trauma experienced in childhood impacted on psychological wellbeing but was generally concealed by the participants as they were fearful of the implications of such disclosure. Although Kieran did well in school and had a supportive group of friends, these childhood events affected his self-esteem and were a source of ongoing distress. He coped by masking and sublimating uncomfortable feelings but the experience of the abuse and the complexity of his feelings for the perpetrator, as well as spending time in care, represented significant, unresolved, issues for Kieran and caused ongoing difficulties in his relationship. As he describes, these issues resurfaced following the breakup of his relationship.

*I think I am very insecure. I put out a great confident attitude but I'm not really. Like everybody that knows me would say yeah I'm very confident and one of my best mates in the hospital the other night said to me you're the last person in the world I'd expect to do it. …*. (I) *Bottle things up*.

***Did you tell anyone about your childhood, about the abuse?***

*I don't know, it's tough. I just wouldn't. I probably would be ashamed of it probably, yeah. I don't think I really have worked it out to be honest with you. I don't think I have*.

***Why do you think?***

*I still don't, not saying nothing was wrong but I didn't see harm in it*.

***Are you ok with that?***

*I shouldn't be okay with it. Maybe I am okay with it but I shouldn't be okay with that. I shouldn't be. I should know that's wrong. I should know like that is totally wrong and if anybody had done that to one of my brothers or sisters or my* (gender of child mentioned) *I would go mad so why am I not going mad because it happened to me. I don't know, I'm just very…. I don't think I have worked it out. I don't think I ever have. I don't think I ever sat down and went through the total story. I have to get over this and I'm not getting over it. …I don't think it's coming from that specific issue. The fact that I spent a year in a children's home and I was going here, there and everywhere. … I wanted to protect him and I didn't want to go home to my mums. I couldn't handle that again. …There was nobody protecting me. It was even there with the whole thing of the break up. …I think the whole thing now with this break-up is that I, remember I told you how I used to go home some weekends and I'd have to go back home, back to the children's home after the weekend and how hard that was for me. Now I'm walking away from her/him* (his child*). I can't see her/him. I don't want to see her/him. I've tried to see her/him over the past three months but I've only seen the child three times. I can't do it. I had her/her out with me yesterday, I had her/him out for a few hours walking around. Crying, just walking around, playing with her/him and then crying. I can't go back or I'll go to bits. I can't do it. Too many bad memories. It's too hard*.

#### Rejection by the father

A number of father-son relationships involved explicit rejection by the father and Dermot's and Fergus's stories are similar in that both concerned their paternity and the father's belief that he was not the man's biological father. Fergus grew up in a home where the communication was gendered, controlled and abusive and where he was a target for his father's anger and rejecting behavior from a young age. He felt powerless to resolve the situation and felt guilty that he was somehow responsible for the rejection. He described a critical event in his life, the occasion when his father told him that he was not his biological father, information which had previously been referred to during alcohol-fuelled outbursts but was now imparted directly to Fergus as he was leaving home to start university.

*Oh I'll never forget that. There's always been a thing. Well not so much lately but when I was younger there was always a thing that my father wasn't my father. Not from my side but from his side*.

***Would he say that to you?***

*Not directly to me but in an argument or whatever with me mam and I'd hear it. There's a long trail there believe me*.

***That goes back to your childhood?***

*More or less yeah. I suppose when I first heard it and I kind of started thinking to myself, I would have been around twelve, thirteen, fourteen years of age. …I always kind of noticed it. Even when I was very young I said the one thing I do before I die is move out of this house before I'm eighteen. There'd be nights when he'd be drunk and I'd hear him slagging me off and saying really really hurtful things. He wouldn't be a violent man toward us. Now he's been violent toward my mam in the past but I shouldn't even say it but it's been drink orientated. There's never been any violence when he was sober. Just a man that's very kind of set in his ways and I was the oldest and I should have been doing this and I should have been doing that. That's half the reason why I didn't want to tell them anything. It's just being the oldest and I didn't want them to be ashamed of me. I was never as close to them as anybody else in the family and we're only a small family, there's only (number mentioned) of us. I was never as close to them as any of the rest of them were. Always about me and never about anybody else, always about me. It's like he had some sort of a vengeance for me. I don't even know to this day whether he truly believes that I'm his son. I loved him but couldn't understand what I needed to do to make him kind of love me. Like I know now that he loves me but all I had to go through to kind of find it out*.

These narratives relate to paternal rejection and the hurt which resulted from this but also demonstrate the son's desire for the father's love and his attempts to salvage what he could from a problematic relationship. Fergus and Dermot hated the abusive behavior directed at them and dealt with the situation by constructing two identities for the father—the good father and the difficult father. This was a common response when the father was abusive as restrictions on expression and the father's status within the home ensured these conflicts were never openly discussed. Dermot felt unable to address the issue of his paternity with his father “as *it might hurt his feelings or whatever*” and Fergus's account of the interaction with his father following his suicide attempt suggests emotional constriction extending over generations, of uncomfortable feelings redirected into anger and violence and the use of alcohol to suppress emotional pain.

***How did your parents respond when they found out?***

*I don't really know but I've only seen my dad for a few hours this morning. He didn't really say much. Me ma came in last night and she was kind of tearful*. (She said) *Me da kind of couldn't understand why we didn't talk to one other*.

***When your father came in what did you talk about?***

*He didn't mention it. He's not good at that kind of thing*.

These stories demonstrate the negative effects on the man's wellbeing of a father's abusive, rejecting, behavior. The participants experienced sadness, shame and self-blame, felt isolated within their families and unable to confide in them. Dermot described his position in the family as “*like the odd one out, well not odd one but kind of left out of things”* and Fergus referred to himself as “*the black sheep*”. Both men hoped that forming intimate partnerships would provide a safe emotional space and help ameliorate these feelings but this did not happen and when the partnerships ended they attempted suicide.

#### Violent fathers

The most hostile father-son conflicts occurred in families where there was physical violence and this was a relatively common occurrence as almost one third of the men were raised in homes where violence was a consistent feature. The mother was usually the victim but some of the men had been subjected to ongoing physical abuse as children. Violence in the home was generally linked to paternal alcoholism and in these instances the participants described childhoods infused with fear and volatility.

*My father was an alcoholic most of my childhood. He'd go from being nice and come home and be someone totally different and then wake up that evening shouting and hitting and kicking. He hit us all. Six nights a week he would come home and be a different person. Just afraid to do anything and I'd just go up to my room. If my sisters were downstairs he'd probably slap them or whatever. The oldest brother left home. I don't know if he ran away or just left home but he left home one night. He used to share the same room and I woke up and the wardrobe was empty and he'd moved*. Ronan

Frank, who was regularly beaten by his father, along with his mother, described a fear-filled childhood and relief when his father left the family home (“*I was just delighted that he left*”). These childhood events affected these men's sense of security and control and made them fearful of pursuing the lives they wanted. Frank was unable to leave an unhappy relationship, which he cited as the precipitant for the suicide attempt, and, as he was reluctant to address issues about his sexuality, refused to engage in therapy. In David's family his father's alcoholism had resulted in violence and a precarious economic situation over many years. As the eldest child David felt a responsibility to protect his mother from his father's violence and his relationship with his father growing up was underpinned by anger but also sadness due to his father's rejecting behavior. Yet, like other participants, he re-engaged with the reformed father although this occurred within the confines of hegemonic constraints on expression.

*There's a bit of friction between the two of us. He used to be an alcoholic and he gave it all up ten years ago. He was pretty difficult. He drank a lot. …Nothing I really like to remember. He was violent to my mother. He's a different person now. He was violent to me a couple of times. I was always in trouble. Every time he came in drunk I was in trouble. She* (mother) *had a hard enough life too. There was never any money there. I remember hating him when I was young. I remember when I was in primary school. I used to come home from school and she might only get dinner once or twice a week because there wouldn't be enough food. She would feed the boys but she wouldn't have eaten for the day. …My father was getting big money at the time from work and it was all going on drink. At the time I remember when he had the problem, when I was going to school I had nothing but cheap clothes. I was very sensitive about that and I'd blame it on him. I was never proud of my father. I'd be proud of him now the way he was an alcoholic and the way he turned himself around. I'd be proud of him now but back then no, I absolutely hated him. …. I hated him and I think he knew that as well*.

***How is your relationship with him since he gave up alcohol?***

*Ok. He's a different person now*.

***Is he the kind of person you could talk to?***

*No. I don't think so, no. I couldn't talk to him anyway. I feel awkward with him. I talk to my mother alright. …Probably because I remember when he used to drink and the fighting. …When I was in the hospital I was told my father was down in the dumps because I hadn't told him. He said that he thought I could talk to him about everything*.

***Have you and your brother ever talked about when you were young***.

*No. I don't even think about when I was young to be honest. …There was a row in the house and someone confronted him about the drink. It nearly killed him. He went out of the room. …Yeah, he just had to go away*.

***It's not something you could discuss with him***.

*I'd never bring that up. It's in the past like you know. He was a different person back then. He wasn't himself*.

Adam had also re-engaged with his father although he had suffered sustained physical abuse from him from an early age, a situation which became normalized for him.

*My father was an alcoholic and he had violent tendencies toward me. He physically abused me as a kid. It was always just me*.

***How often would that happen?***

*Once every few days*

***And how did that make you feel?***

*Afraid. …I suppose resentful I guess now that I have gotten a bit older. I still talk to him.…. It wasn't entirely his fault, he's manic depressive and he's an alcoholic. I don't think it's entirely his fault*.

***But at the time?***

*I was terrified*.

***You feel differently about him now?***

*Yeah, I feel sorry for him*.

***When you were growing up, did you have someone to talk to about your problems?***

*No*.

***Did you ever tell anyone?***

*No*.

***Did you talk to your mum?***

*No*.

***Did you have any close friends at that time?***

*A few, yeah, but I didn't talk to them about it because I thought it was normal, I thought it was like that in every house. I didn't think it was abnormal. When you grow up with things you think they're normal, it's only when you start watching television it seems that like* (they are not).

Men who experienced an abusive environment as children recounted the long-term effects of this on their self-esteem and many, particularly those who were victimized in school, described a kind of layering or build-up of distress over time. The adversity experienced by these men would be challenging for any individuals but their situation was compounded by the lack of an outlet to speak about these events as they felt unable to do so due to gendered restrictions on expression. Confiding in fathers was not an option for these men and other family members and male friends were regarded as equally inaccessible (Cleary, 2019). In contrast to traditional accounts of male and female psychological processes, painful feelings were internalized and only rarely directed outwardly *via* violent behavior. A background narrative in these stories was the generational transmission of hegemonic masculinity cultures and their effects. Participants' accounts of their fathers' behavior suggested that they lacked the ability to engage meaningfully with their sons and did not have a language to express nurturing feelings. The evident unhappiness of many fathers, implied by the men's accounts of their fathers' behavior and alcoholism, appeared to represent the transmission of this unhappiness as well as deficient models of masculinity into the next generation.

*His father abused him for years… …I'd say my father was asked to go for help. I'd say he was. He had to be told he needed help at some stage in his life and I think that he did probably at one stage maybe try and didn't like it or something, and that was it*. Sean

## Discussion

Linkages between practices of hegemonic masculinity, particularly emotional constraint, and male suicide have been identified in the research literature (Cleary, 2012, 2019) and the aim of this study was to explore the implications of exposure to family and neighborhood cultures of feeling restriction and the contribution of fathers and father-son relations to these environments. The study, based on a sample of men who made a clinically serious suicide attempt in adulthood, illustrated how the masculinity scripts available to these men, who were predominantly from low socioeconomic backgrounds, defined manhood in narrow, conventional, terms, emphasizing strength and discouraging expressions of vulnerability. The theme *learning about masculinity* revealed how the culture of masculinity and emotional expression these men were exposed to growing up shaped their ideas about gender and what were acceptable practices for males. This masculinity framework was contested and the men's accounts suggest that adherence to these gender norms was more accurately about public display while in private they held more flexible views. They felt pressure to conform due to expectations within the family and, as revealed by the theme *the regulation and enforcement of behavior by* peers, they feared the consequences of diverging from hegemonic ideals in the neighborhood. Bullying was used extensively in schools to enforce conformity to these practices and they were largely confined to these areas growing up due to socioeconomic factors. In this way hegemonic principles became the baseline and working model for the men's masculinity performances and socioeconomic factors constricted their ability to explore alternative possibilities, as described elsewhere (Redley, 2003; Turner et al., 2006).

Fathers were significant figures in these men's lives and were instrumental in providing and scrutinizing early and ongoing markers of masculinity. The emotional culture within the home was gendered, in line with hegemonic ideals, and there were distinct male and female expressive and interaction styles. This gendered emotional culture shaped male behavior and relations within the home, including father-son interaction, and inhibited the communication of feeling needs and the development of nurturing bonds between fathers and sons. The third theme (*relationships with fathers*) demonstrated the influence of the father on the man's practice of masculinity and the implications of negative father-son relationships for both gender and wellbeing. The theme s*eeking love and care from the father* indicated that the participants sought an intimate connection with their fathers but relations with fathers, whether they lived within or outside the home, were lacking in emotional closeness, almost never harmonious and more commonly antagonistic. Fathers were generally perceived as emotionally distant, as unable or unwilling to express love and care and some fathers were rejecting and abusive toward their sons. These findings support other research work which indicates that hegemonic masculinity principles are a barrier to close, nurturing, father-son relationships (DeFranc and Mahalik, 2002). While a father is not essential for a boy's wellbeing (Pleck, 2010) nurturing father-son relationships are beneficial for the young male's emotional development and mental health (Lamb, 2010; Adamsons and Johnson, 2013). In this study, fathers adhered to hegemonic principles and there were almost no examples of fathers who openly and consistently nurtured their sons. The intergenerational transmission of deficient models of emotional communication and father-son relationships, identified elsewhere (Brown et al., 2018; Jessee and Adamsons, 2018), was also apparent in this study.

As in other studies of male suicidal behavior, many of these men were exposed to ill-treatment and trauma in childhood and this issue is explored in the themes *rejection by the father* and v*iolent fathers*. Paternal violence and alcoholism was relatively common and this caused suffering and anger and limited the participants' emotional, social and economic lives as they grew. Yet, these men did not replicate the father's violence and sought to salvage something from these relationships and connect with their fathers. Growing up in a problematic family resulted in emotional and physical suffering and affected their psychological security from a young age and this is supported by similar findings (Molnar et al., 2001; Seguin et al., 2011; Hughes et al., 2017; Giupponi et al., 2018). The type of violence they experienced impacts on one's sense of security and control and is associated with suicidal behavior (Dube et al., 2001; Enns et al., 2006). Difficulties encountered at home frequently led to other problems such as victimization in school and the result was a build-up of distress over time which has implications for mental health (Oliffe et al., 2021). Adverse childhood experiences require an appropriate response to avoid negative outcomes (Seguin et al., 2011; Hughes et al., 2017; Giupponi et al., 2018) but constraints on emotional expression and shame prevented the participants from speaking about their suffering and this exacerbated their situation and made it more likely that the psychological burden was carried into adulthood. As in Ridge et al.'s (2020) study the men lacked self-esteem, felt they had under-achieved in academic and career terms and recounted ongoing distress and situations which rekindled memories and unsettled their security. They coped by denial and rationalization, by self-medicating, and by seeking emotional solace within intimate partnerships. These patterns have been reported elsewhere in the literature (Afifi et al., 2009; Hughes et al., 2017) but self-medication is likely to aggravate these situations (Cleary, 2019) and, as Oliffe et al. (2022) have also demonstrated, the complexities these men brought to partnerships tended to destabilize them and they were especially vulnerable when relationships ended. These findings, which add detail to the processes and impact of emotional constraint, may help to explain the higher risk of suicide for males, compared to females, following childhood trauma (Dube et al., 2001; Wagner et al., 2003; Afifi et al., 2009; Weich et al., 2009) and more generally, provide insights in terms of higher male, compared to female, rates of suicide.

## Conclusion

This study, based on a sample of men who made a clinically serious suicide attempt in adulthood, revealed how exposure to a hegemonic form of masculinity from an early age influenced the men's gender practices and their wellbeing. The results illustrate how hegemonic type masculinity cultures restrict males in learning about and negotiating the emotional issues of their lives and the importance of family, fathers, and neighborhood in the development and continuance of these gender practices. The participants felt pressure to conform to the prevailing masculinity due to expectations within the family and the enforcement of these practices in the neighborhood and socioeconomic factors constricted their ability to explore alternative gender possibilities. In these circumstances, this form of masculinity became a working model for the men's masculinity performances but this does not imply the homogeneity of male behavior and experiences even within particular communities. Men do not have identical lives nor experiences and life trajectories are variable whatever the personal, cultural, or structural background. However, these results indicate that a specific combination of experiences and situations create a higher probability of prolonging distress and layering problems over time. The masculinity culture they inhabited required them to suppress particular emotions, disguise vulnerabilities and conceal distress and this entailed a psychological burden over time especially for those men who had encountered trauma growing up. The experiences of these men are important in understanding the motives of those who attempt suicide as they provide detail on how emotional constraint can impact on young males, particularly those with restricted social and economic options. They were capable of discussing their emotional lives in considerable detail as demonstrated at interview and this, along with the wide array of feelings conveyed, implies a facility to address these issues as well as refuting binary ideas about emotions (Shields, 2007; Patulny et al., 2017). The results suggest a need to re-examine gender barriers to disclosure and treatment (Cleary, 2017) and to replace binary and singular ideas about men's emotions with more realistic concepts in suicide prevention programs. The findings, which indicate the importance of fathers to male lives and wellbeing imply a requirement to incorporate the benefits of emotionally engaged and affirming fathering into Public Health campaigns and discussions. More specifically, health providers might provide upskilling in communication for parents, especially fathers, to assist them in the aftermath of a son's suicide attempt.

## Data availability statement

The qualitative data presented in this article are not available due to confidentiality requirements. Requests for further information should be directed to anne.cleary@ucd.ie.

## Ethics statement

The studies involving human participants were reviewed and approved by the ethics committees of the participating hospitals. The patients/participants provided their written informed consent to participate in this study.

## Author contributions

AC conceptualized and developed the study, conducted all interviews, analyzed the data, and wrote the paper.

## Funding

This study was supported by Irish Research Council.

## Conflict of interest

The author declares that the research was conducted in the absence of any commercial or financial relationships that could be construed as a potential conflict of interest.

## Publisher's note

All claims expressed in this article are solely those of the authors and do not necessarily represent those of their affiliated organizations, or those of the publisher, the editors and the reviewers. Any product that may be evaluated in this article, or claim that may be made by its manufacturer, is not guaranteed or endorsed by the publisher.
